# Vascular restoration therapy and bioresorbable vascular scaffold

**DOI:** 10.1093/rb/rbu005

**Published:** 2014-10-20

**Authors:** Yunbing Wang, Xingdong Zhang

**Affiliations:** National Engineering Research Center For Biomaterials, Sichuan University, Chengdu 610064, China

**Keywords:** bioresorbable vascular scaffold, vascular restoration therapy, vascular reparative therapy, vascular disease, bioresorbable/biodegradable material, drug-eluting stent, drug-eluting biodegradable stent, drug-eluting bioresorbable scaffold

## Abstract

This article describes the evolution of minimally invasive intervention technologies for vascular restoration therapy from early-stage balloon angioplasty in 1970s, metallic bare metal stent and metallic drug-eluting stent technologies in 1990s and 2000s, to bioresorbable vascular scaffold (BVS) technology in large-scale development in recent years. The history, the current stage, the challenges and the future of BVS development are discussed in detail as the best available approach for vascular restoration therapy. The criteria of materials selection, design and processing principles of BVS, and the corresponding clinical trial results are also summarized in this article.

## Introduction

Vascular disease is a pathological state of muscular arteries. It starts with endothelial cell dysfunction, which is followed by the proliferation and migration of smooth muscle cells (SMCs) toward the blood vessel lumen. Such chronic effect causes thickening of the vessel wall, forming a plaque due to the build-up of fat and cholesterol deposits on the inside walls consisting of proliferating SMCs, macrophages and various types of lymphocytes. Over time, the build-up narrows the artery and causes inadequate blood flow to various body tissues. Eventually, the plaque may also rupture causing the formation of clots. The blockage formed in the coronary arteries could potentially cause angina or even heart attack. When the blockage forms in the carotid arteries, it can lead to a transient ischemic attack or even stroke. When the blockage forms in the legs, it could lead to leg pain or cramps with activity, while a total loss of circulation can even cause gangrene and loss of a limb.

Among various vascular diseases, the cardiovascular disease, especially coronary artery disease (CAD), is by far the leading cause of mortality worldwide. Currently, around 17 million people are dying of this disease worldwide each year, and this number is expected to exceed 23 million by the year 2030 [1,2].

In order to treat these vascular diseases, the development of a new therapy/device has been long expected, which could help the narrowed vessel restore its original size to re-establish its normal blood flow condition, regain the once damaged endothelial monolayer to prevent the formation of plaque and recover its normal physiological vascular function. Ultimately, it is also expected that the new therapy/device could even demolish or eliminate the formed plague and allow the diseased vessel to regain its original healthy conditions. Such approach has been widely discussed as vascular restoration therapy or vascular reparative therapy [3–14].

## Previous approaches for vascular restoration

Originally, various efforts had been done to develop medicines which could repair the diseased vessel and restore its healthy function. But so far, most of them normally can only temporarily relieve the symptom of the disease and could hardly play the magic to move the narrowed vessel back to its original size.

In early 1960s, the by-pass surgery was introduced for CAD treatment. However, such open heart surgery was very complicated and required long time to be fully recovered [15].

In 1970s, the percutaneous transluminal coronary angioplasty (PTCA), or simply called as balloon angioplasty, was developed aimed at restoring the regular blood flow to repair diseased vessel. The PTCA is performed by advancing a small guide wire across the blockage in the blood vessel first, followed by advancing a pre-crimped balloon to the blockage site through the guide wire. Once the pre-crimped balloon arrives at the blockage site, it is inflated to compress the blockage against the artery wall to open up blocked coronary artery, allowing blood to circulate unobstructed to the heart muscle. At the end of the procedure, the balloon is deflated and withdrawn from the blood vessel. Since the first performance of PTCA by Dr. Gruentzig in 1977 in patients [16,17], it had been quickly applied for cardiovascular disease treatment around the world. This revolutionary technology is viewed as the initial step toward vascular restoration therapy. However, due to the lack of support to keep the vessel open once the balloon was deflated, the high restenosis (30–50%) due to elastic recoil, constrictive remodeling and intimal hyperplasia were observed as the common side-effects of balloon angioplasty, which resulted in high rate of repeat interventions at long-term follow-up, representing a limitation of such technology [18].

In order to prevent restenosis due to elastic recoil and constrictive remodeling of diseased vessel by balloon angioplasty, bare metal stent (BMS), which is a small tubular wire mesh device, was developed and quickly widely applied around the world. In practice, BMS is crimped onto a balloon catheter first. Once it is expanded in the narrowed section of the vessel, it maintains post-treatment vessel patency and prevents elastic recoil and constrictive remodeling [19,20]. Palmaz-Schatz balloon-expandable stent was approved in USA as the first BMS for elective use by Food and Drug Administration (FDA) in 1994.

Compared to expanded balloon, BMS could keep the narrowed vessel open to re-establish its normal blood flow condition. It seemed that BMS technology might be one step closer to serve as vascular restoration therapy. However, after large-scale application of BMS in 1990s, it was found that although BMS reduced rates of restenosis compared with balloon angioplasty, the re-narrowing of the treated artery was still observed in 20–30% of patients due to in-stent restenosis [21]. The in-stent restenosis is defined as diameter stenosis of ≥50% in the stented area of the vessel mainly due to excessive neointimal proliferation within the stented segment initiated by inflammatory response in the vessel wall area with implanted BMS, where the inward migration and proliferation of medial SMCs and the deposition of excess extracellular matrix proteins would ultimately obstruct the vessel lumen [22–25].

In order to reduce in-stent restenosis caused by BMS, drug-eluting stent (DES) was developed in late 1990s [26–30]. DES normally contains three components: stent platform, therapeutic agents/drug and drug carrier. The stent platform ensures the patency of the vessel lumen, whereas the drug and drug carrier-formed coating layer controls the drug release to limit the growth of neointimal scar tissue, thus reducing in-stent restenosis. For drug carrier, it is required to have good adhesion with stent platform, good bio-compatibility, good mechanical properties such as good strength and elasticity. Polymer materials have been used as suitable candidates of drug carriers from the beginning. By adjusting the drug/drug carrier composition, the drug dose and drug-releasing kinetics could be well controlled.

DES stent platform can be made from the following materials: 316L stainless steel, cobalt–chromium alloy, titanium and its alloy, platinum–iridium alloy and tantalum, etc.

Many therapeutic agents with antiproliferative and/or anti-inflammatory properties, such as everolimus, sirolimus, zotarolimus, biolimus, tacrolimus and paclitaxel have been incorporated on the stent surface and tested clinically to inhibit neointimal growth [31].

The first successful trial of DES was of sirolimus-eluting stent. A clinical trial conducted in 2002 led to regulatory approval of the sirolimus-eluting Cypher™ stent manufactured by J&J Company in Europe in 2002, and its FDA approval was obtained in 2003. Followed by the approval of Cypher™ stent, the paclitaxel-eluting Taxus™ stent was manufactured by Boston Scientific and obtained FDA approval in 2004. In addition, the zotarolimus-eluting Endeavor™ stent manufactured by Medtronic and everolimus-eluting Xience V™ stent manufactured by Abbott were approved by FDA in 2007 and 2008, respectively.

Although the DES could greatly reduce the in-stent restenosis, the continuous release of drug could cause the delay of artery healing process, and potentially increase the risks of late stent thrombosis after discontinuation of antiplatelet therapy. In case of DES with durable polymer as coating material, especially for DES with less biocompatible durable polymer coating layer, the durable polymer remaining within the coronary artery environment long after complete elution of drug may contribute to the adhesion and activation of leukocytes, leading to the local chronic inflammation and hypersensitivity, which could potentially increase the risks of late stent thrombosis [32,33] and late in-stent restenosis [34]. In order to eliminate the potential effect of durable polymer on late stent thrombosis and late in-stent restenosis, many studies focusing on DES with biodegradable polymer coating layer have been conducted [35–49].

For current regular metallic DES, though its coating layer is made of durable polymer or biodegradable polymer, the backbone platform is a non-degradable metal, which stays in the vessel permanently after implantation, which makes any further non-invasive screening or re-intervention more difficult, and would also potentially cause late thrombosis and late in-stent restenosis after the anti-restenosis drug is completely released. In addition, irrespective of the kind of coating layer is used, as long as the stent platform still has biostable metal, the vessel would not be able to fully regain its original physiological functions such as vasomotion functions.

## Bioresorbable Vascular Scaffold

Bioresorbable vascular scaffold (or fully biodegradable stent) is a small tubular wire mesh device with its platform made from fully bioresorbable materials, which normally has a coating layer containing bioresorbable material and anti-proliferative drug on the top of its platform like regular DES. Bioresorbable vascular scaffold (BVS) is used to avoid permanent metal implant in vessels, allow late vessel remodeling in the absence of a metallic cage and leave only healed native vessel tissue after the full absorption of the scaffold. The stented segment might recover its healthy condition, allowing physiological vascular function after re-establishing normal flow conditions by lumen expansion.

Compared to regular DES, BVS could reduce the possibility of additional interventions at the sites of device implantation without concerns for the generation of jailed side branches and does not interfere with non-invasive diagnostic tools [50–52]. The lack of a foreign object in the body might reduce the risk of potential long-term complications and of late thrombosis. The bioresorbable vascular scaffold has the potential to restore vascular integrity and fulfill all requirements of vascular restoration therapy.

In the early stage of development of BVS back to 1980s, none of them contained anti-proliferative drug coating layer. Nowadays, most of the bioresorbable vascular vcaffolds are designed to have drug coating layer to perform all the functions of a DES first and then are naturally absorbed and metabolized in the body. The absence of permanent implant would eliminate the stimulus for chronic inflammation and accelerate healing process, and potentially reduce the need for long-term dual anti-platelet therapy. Late stent thrombosis is thereby abolished or greatly reduced. In addition, the disappearance of the bioresorbable vascular scaffold facilitates any re-intervention at later stages when needed [53,54].

### Principles of BVS design and processing

Since early 1980s, a lot of R&D work on BVS has been done in both academic and industrial areas and some major breakthroughs have been achieved in the recent 10 years [54–62]. Based on the recent studies and various data generated from bench testing, preclinical and clinical studies, it has been found that the BVS design should meet a different set of performance criteria other than regular metallic DES design. The lifecycle of a BVS contains three phases: (i) revascularization of blocked vessel after scaffold implantation; (ii) restoration of reopened vessel; and (iii) resorption of BVS. During the first phase, the BVS should have high acute radial strength, minimum acute recoil, good deliverability and therapeutic agent delivered to abluminal tissue at a controlled rate. During the second phase, the BVS should gradually lose radial strength, deposit cellular matrix over struts and allow the vessel to respond naturally to physiological stimuli. In the third phase, the scaffold should become discontinuous structure and be absorbed in a benign fashion [63,64]. In order to open the blocked vessel and provide mechanical support, the BVS must have high radial strength and a certain degree of flexibility. In addition, the degradation products need to be fully non-toxic. Given an ideal design pattern and processing conditions, the BVS must be able to perform its mechanical function, facilitate scaffold placement within the vessel and deliver drugs for the prevention of restenosis. Furthermore, the scaffold must support the artery while the vessel heals and should gradually transfer mechanical load to the tissue as the scaffold degrades over time. Radiopacity is another consideration in BVS design. The scaffold should be visible under X-ray while allowing visualization of the vessel and balloon catheter markers. In order to achieve success, a BVS is expected to have comparable efficacy to a DES while absorbs safely over a clinically appropriate time frame.

### Materials for bioresorbable vascular scaffold preparation

In order to obtain qualified product of bioresorbable vascular scaffold, there are some basic criteria for material selection. First, a candidate material for BVS should possess enough mechanical strength to hold open the blocked vessel and provide mechanical support without significant recoil, while the candidate should also have a certain degree of fracture toughness to avoid cracks or broken struts. Then, the resorption rate of candidate material should match the healing process of blood vessel, and the degradation products should not provoke tissue overload and other inflammatory responses. And then, the material should have great biocompatibility, good fatigue resistance and physical resistance to aging. At last, the material should be able to withstand high-temperature heat processing, high-energy laser cutting and critical sterilization conditions.

Materials that have potential to be used in bioresorbable vascular scaffold preparation include various bioresorbable polymeric materials and blends [54, 65–77], and corrosive metallic metals and alloys [78–93]. Representative bioresorbable materials which might be potentially used for BVS preparation are briefly summarized in Table 1. In addition, there are also researches on bioresorbable vascular scaffold made from hybrid bioresirbable materials such as composites of bioresorbable polymer/bioceramic, and bioresorbable metal/bioceramic [94,95].

**Table 1.** Representative bioresorbable materials which could potentially be used for bioresorbable vascular scaffold preparation

Name	Degradation time(months)
Poly(D,L-lactide)	>12
Poly(L-lactide)	>24
Polyglycolide	6–12
Poly(lactide-co-glycolide)	1–28
Poly(ε-caprolactone)	>24
Poly(trimethylene carbonate)	>12
Poly(anhydride)	1–6
Poly(urethane)	>6
Magnesium	<3
Iron	>24

Bioresorbable polymeric materials include bioresorbable polyesters [54, 65–73], polyanhydrides, polyurethanes [74], polyorthoesters, poly(ester amide) [75], poly(amino acid) and tyrosine-derived polycarbonates [76,77]. Among these polymers, polyesters have been used in a wide range of medical devices. The typical examples of polyesters include poly(L-lactide) (PLLA), (poly-D-lactide) (PDLA), poly(D,L-lactide) (PDLLA), polyglycolide (PGA), poly(ε-caprolactone) (PCL), poly(trimethylene carbonate), and their copolymers such as poly(lactide-co-glycolide) and poly(lactide-co-caprolactone), etc. [65–68]. PLLA comprises the naturally occurring (L) enantiomer of the lactide monomer. The PLLA homopolymer is semicrystalline, whereas the PDLLA polymer is amorphous due to the racemic mixture of monomer that disrupts cystallinity. As a result, PDLLA erodes at a faster rate than PLLA, which normally degrades over a few years. On the other hand, PGA is a highly crystalline polymer, but its bonds are highly prone to hydrolysis. PGA homopolymer typically degrades over a time period of 6–12 months. Copolymer of 50:50 PGA and PLA degrades more rapidly than either homopolymer because the PLA interrupts the crystallinity of PGA and allows for more water penetration. Homopolymer and copolymers mentioned above can also be mixed together to form various blends such as PLLA/PCL blend, PLLA/PGA/PCL blend, PLLA/PDLA stereo-complex blend.

As for corrosive metals, most studies have been focused on the following two classes of metals: magnesium (Mg) and iron (Fe) and Mg- or Fe-based alloys. The Fe and Fe-based alloys include pure Fe [78–81], nitride Fe [82–83], and Fe–Mn alloys [84–87], etc. Mg-based alloys under investigation include magnesium–rare earth (Mg–RE) [88–90], magnesium–aluminum (Mg–Al) [91,92], Mg–Zn and magnesium–calcium (Mg–Ca) alloy [93], etc.

In spite of availability of quite a lot of bioresorbable materials, the number of materials which would meet all requirements mentioned above at the same time is limited. Among them, PLLA has been used as the most popular material for various BVS products development.

Based on clinical trial results, the bioresorbable vascular polymeric scaffold, especially PLLA-based scaffold, has shown very promising results and some products have received CE marking approval. As for bioresorbable vascular metallic scaffold, no product has been approved for sale in the market. For PLLA-based scaffold, during degradation, the polymer breaks down into lactic acid and is ultimately metabolized into water and carbon dioxide. As for bioresorbable vascular metallic scaffold, the case could potentially be more complicated partially due to continuous release of corrosive product.

### Various BVSs under development

Since 1980s, especially in recent 10 years, dozens of medical device companies in the world have been developing various BVS products, which are listed in Table 2.

**Table 2.** Bioresorbable vascular scaffold products under development

Company	Stent name	Platform material	Drug	Coating material
Abbott	Absorb™	PLLA	Everolimus	PLA
REVA Medical	ReZolve®	Tyrosine-derivedpolycarbonate	Sirolimus	Absorbable polymer
Elixir	DESolve™	PLLA	Myolimus	PLA
Kyoto Medical	Igaki–Tamai	PLLA	Nil	Nil
Amaranth Medical	Amaranth PLLA	PLLA	Nil	Nil
Huaan Biotech	Xinsorb	PLLA	Sirolimus	PLA
OrbusNeich	Acute	PLLA-based polymer	Sirolimus/CD34	Absorbable polymer
Arterial Remolding Technologies	ARTDIVA	PLLA	Nil	Nil
Xenogenics Corporation	Ideal	PAE salicylic acid	Sirolimus salicylate	Salicylate linked by adipic acid
Biotronik	AMS I	WE43 Mg alloy	Nil	Nil
	DREAMS I	Refined Mg alloy	Paclitaxel	PLGA
	DREAMS II	Refined Mg alloy	Sirolimus	PLA
Lifetech Scientific	Nitriding iron stent	Nitride iron	Nil	Nil

The first BVS prototype was developed by researchers from Duke University in the early 1980s, and was based on PLLA. The scaffold was described as self-expanding. The preclinical study was performed with five stents and pathologic data were obtained at various time-points between 2 hours and 12 weeks. No inflammatory response and no thrombosis were observed. It was reported that the stents were endothelialized after 2 weeks [96,97].

The first BVS in clinical trial was Igaki–Tamai BVS/stent, which was manufactured from a monofilament poly(L-lactide) fiber and wound into a helical pattern [52, 98–100]. The scaffold was designed as a ‘zigzag’ coil and was described as springy and partially self-expanding. It requires delivery in a covered sheath (8F), and once positioned, deployment requires balloon expansion with contrast heated to as high as more than 55°C. Subsequent self-expansion occurs over a 30-min period [100].

In 1998, an unblinded clinical study on 55 patients was undertaken to assess the feasibility and safety of the Igaki–Tamai scaffold/stent in coronary arteries, and were followed up to 4 years [101–103]. A total of 84 stents were used to treat 63 lesions. X-ray angiography and intravascular ultrasound (IVUS) were performed before and immediately after the procedure to visualize results, and up to 48 months following the initial procedure. The clinical studies showed that all stents were successfully delivered to the target lesions and that angiographic success was achieved in all procedures performed. Immediately after the procedure, angiography and IVUS revealed no further bioactive remodeling of the stented segment. At 12-month post-procedure IVUS follow-up, the scaffold struts were clearly visible and apparently well opposed to the vessel wall. IVUS measurement taken 36 months post-procedure showed a decrease in scaffold strut area. Overall, the Igaki–Tamai biodegradable scaffold/stent study demonstrated the feasibility and safety of the BVS/stent over a 4-year follow-up period.

In 2012, the 10-year follow-up data from a second larger cohort of 50 patients were reported. The cumulative target lesion revascularization over 10 years was 28%, which was comparable to BMS [104].

One concern of using Igaki–Tamai scaffold/stent is expansion of the stent due to heat, because the exposure to elevated temperature can cause necrosis of the arterial wall, followed by SMC proliferation [105]. A temperature of 55°C has been shown to promote platelet adhesion to the vessel wall, and thus the increase of thrombosis [100].

Reva Medical Inc. is a company developing bioresorbable scaffold from a radiopaque tyrosine-derived polycarbonate material and has initiated its clinical trial since 2007. Reva Medical utilizes a unique ‘slide and lock’ geometry for its scaffold designs. When expanded, the stent elements slide from the compact state and lock into an expanded state, similar to safety lockouts on extension ladders. Thus, the expansion is not dependent on material deformation, and will provide steel-like scaffolding. The Reva BVS is delivered by standard balloon deployment and is made radiopaque by polymeric material itself.

Among various metallic BVSs, Biotronik (Berlin, Germany) is the first and also the only company who has product under clinical trial stage used in human coronary arteries so far. Biotronik's first generation metallic BVS is made from magnesium alloy WE43 containing 93% magnesium and 7% rare earth metals. Its First-in-Man clinical trial showed that its late lumen loss at 4 months was unacceptably high (1.08 ± 0.49mm) and its ischemic-driven Target lesion revascularization (TLR) rate reached to as high as 26.7% after at 12 months, which was possibly caused due to too fast degradation (it degraded within 2 months) [106]. With the modification of the composition of the magnesium alloy and the addition of PLG/drug coating layer, the second generation product showed considerable improvement with the late lumen loss at 0.64 ± 0.50 mm at 6 months follow-up and the TLR rate down to 4.7% at 12 months [107].

### ABSORB BVS

The Abbott ABSORB BVS is the first drug-eluting BVS with CE marking approval. Back in 2006, Abbott initiated First-in-Man ABSORB clinical trial, the world’s first clinical trial of drug-eluting polymeric BVS [108]. The ABSORB BVS has been available in Europe and other international markets since late 2012, and a large-scale clinical trial of ABSORB BVS has started since 2013 in USA.

Like the Duke and Igaki–Tamai scaffolds, the Abbott ABSORB BVS is also made from PLLA. However, Abbott ABSORB BVS has a coating layer containing antiproliferative agent everolimus and polylactide to control drug release. The scaffold is made radio-opaque by the addition of radial-opaque markers on the two ends of the scaffold. The acute property of ABSORB BVS after deployment looks similar to regular DES with good mechanical properties, deliverability and biocompatibility. The ABSORB BVS has similar radial strength as Abbott MULTI-LINK metallic stent. The *in vivo* preclinical model of the drug release profile from ABSORB BVS is comparable to Abbott’s XIENCE™ V everolimus drug-eluting coronary stent, which has been approved for use in Europe and USA.

A 2-year pre-clinical study of ABSORB BVS showed that BVS was safe and effective. Complete luminal endothelialization was formed within 1 month. Similar to Cypher DES, the neointimal response of BVS is minimal at all time points evaluated up to 12 months. The inflammatory response of BVS is overall minimal and is even less than that of the Cypher DES at 6 and 9 months [109,110].

The outcome of 2-year follow-up from the ABSORB clinical trial indicated the full absorption of scaffold. Unique benefits such as lumen gain and restored vasomotion have been demonstrated clinically after treatment with ABSORB BVS [111]. The late lumen enlargement was associated with reduced plaque burden and vasomotion was restored similar to the native state of a coronary artery [112]. Absence of foreign body reaction and the recovery of vasomotion restoration were indications of a healthy vessel and suggested that late thrombosis risk had been eliminated. Compared to SPIRIT I clinical trial results using regular DES, after 2 years, the ABSORB BVS exhibits positive remodeling and full resorption of the scaffold and a trend back to the deployment state. Such trends are not observed with non-degradable DES since there are permanent scaffolding effects preventing the lumen from exhibiting positive remodeling. These results suggest that the BVS is trending back to its healthy native state.

## The future of BVS

The clinical trial results with BVS, especially with Abbott ABSORB BVS, have shown that BVS could let the narrowed vessel to re-establish its normal blood flow condition. Furthermore, it could even recover the normal physiological vascular functions in some cases, which makes BVS the best available approach for vascular restoration therapy.

It is also expected that the large-scale application of BVS would dramatically shorten the dual-antiplatelet therapy duration and possibly remove the risk of late thrombotic events associated with a permanent scaffold.

In the near future, it requires more research work to further optimize BVS material design and processing to be sure of its bioresorption profile would match the vessel healing process very well. By doing so, it is expected that more diseased vessels would regain their normal physiological vascular functions after vascular restoration therapy using BVS.

Another optimization work to do in the future is to increase the rate of full re-endothelialization to promote adequate vessel healing. Although the healthy animal model showed that the re-endothelialization of BVS can be finished within 1 month, but based on clinical results from regular DES, it was known that the continuous release of antiproliferative drug would delay or even inhibit the formation of a complete endothelial mono-layer. Such delay or inhibition would definitely impact the recovery of normal physiological vascular functions.

The vascular healing processes are expected to be very complex and may vary from patient to patient. Therefore, in the future, there is a demand to make BVS product with different materials and different designs to meet different requirement.

It is anticipated that BVSs offer the possibility for integration with local drug delivery, genetic transfer and radiation. Geometrically modified fully biodegradable implants can act as potential therapeutic carriers for the treatment of diseases such as cancers, where specific tissue targeting and high local dosage possibilities could offer an alternative to conventional systemic therapies.

Finally, it is expected that the success of BVSs applied in coronary arteries will also prompt the expansion to peripheral arteries, the trachea, esophagus, and urethra, etc. in the future.
